# Preemptive interleukin-6 blockade in patients with COVID-19

**DOI:** 10.1038/s41598-020-74001-3

**Published:** 2020-10-08

**Authors:** Lucía Guillén, Sergio Padilla, Marta Fernández, Vanesa Agulló, José Alberto García, Guillermo Telenti, Javier García-Abellán, Ángela Botella, Félix Gutiérrez, Mar Masiá

**Affiliations:** 1 Infectious Diseases Unit, Hospital General Universitario de Elche, Camí de la Almazara S/N, Elche, 03203 Alicante, Spain; 2 Clinical Medicine Department, Universidad Miguel Hernández, Ctra. de Valencia (N-322), Km 87, 03550 San Juan de Alicante, Spain; 3 Universidad Miguel Hernández, Avda de la Universidad S/N, Elche, 03202 Alicante, Spain

**Keywords:** Infectious diseases, Health care

## Abstract

Excessive interleukin-6 signaling is a key factor contributing to the cytokine release syndrome implicated in clinical manifestations of COVID-19. Preliminary results suggest that tocilizumab, a humanized monoclonal anti-interleukin-6 receptor antibody, may be beneficial in severely ill patients, but no data are available on earlier stages of disease. An anticipated blockade of interleukin-6 might hypothetically prevent the catastrophic consequences of the overt cytokine storm. We evaluated early-given tocilizumab in patients hospitalized with COVID-19, and identified outcome predictors. Consecutive patients with initial Sequential-Organ-Failure-Assessment (SOFA) score < 3 fulfilling pre-defined criteria were treated with tocilizumab. Serial plasma biomarkers and nasopharyngeal swabs were collected. Of 193 patients admitted with COVID-19, 64 met the inclusion criteria. After tocilizumab, 49 (76.6%) had an early favorable response. Adjusted predictors of response were gender, SOFA score, neutrophil/lymphocyte ratio, Charlson comorbidity index and systolic blood pressure. At week-4, 56.1% of responders and 30% of non-responders had cleared the SARS-CoV-2 from nasopharynx. Temporal profiles of interleukin-6, C-reactive protein, neutrophil/lymphocyte ratio, NT-ProBNP, D-dimer, and cardiac-troponin-I differed according to tocilizumab response and discriminated final in-hospital outcome. No deaths or disease recurrences were observed. Preemptive therapy with tocilizumab was safe and associated with favorable outcomes in most patients. Biological and clinical markers predicted outcomes.

## Introduction

The pandemic of coronavirus disease 2019 (COVID-19) has become one of the greatest global health challenges. The novel severe acute respiratory syndrome coronavirus-2 (SARS-CoV-2) has spread to the five continents and caused more than 8,000,000 new infections and 400,000 deaths. Although the majority of infected people have a mild disease with good prognosis, a subset of patients develops bilateral interstitial pneumonia, diffuse alveolar injury and acute respiratory distress syndrome (ARDS), which carries a mortality higher than 50% and constitutes the main cause of death^1,2^. In contrast to other respiratory viral infections like influenza, a major pathogenic mechanism implicated in severe clinical manifestations of COVID-19 is an aberrant host immune response resulting in an excessive cytokine and chemokine release known as “cytokine storm” or “cytokine release syndrome”^2,3^. Among immune molecules contributing to the cytokine-mediated damage and inflammation, interleukin-6 (IL-6) is one of the key factors^3^. Patients with COVID-19 show high plasma levels of IL-6, which correlate with disease severity and multiorgan failure^4,5^. Excessive IL-6 signaling induces different biological effects that, in addition to the immune system and inflammation mediators, also involve the blood vessels or the myocardium, and result in organ damage^6,7^. Due to the different pathogenic mechanisms implicated in COVID-19, combined therapeutic approaches including both antivirals and blockage of inflammatory pathways have been advocated for an optimal disease control^8^.

Tocilizumab (TCZ) is a humanized monoclonal anti-IL-6 receptor antibody, subclass IgG1. Because of the prominent role of IL-6 in COVID-19, it was the first drug proposed for the treatment of the inflammatory complications in patients with severe disease^9^. However, data are limited to a scarce number of studies that mostly included severely ill patients^10–13^. Several questions remain to be elucidated. First, whether TCZ administration at an earlier stage of disease might hypothetically prevent the catastrophic consequences of the overt cytokine storm. As a counterpart, since IL-6 can both facilitate but also suppress viral replication^14,15^, TCZ could have a detrimental impact on viral clearance if given prematurely. Therefore, information on the effect of TCZ on SARS-CoV-2 replication might help understanding the benefits and risks of therapy. A third question refers to whether there could be predictors of a higher probability of response to TCZ to select the most suitable candidates for therapy and to design alternative strategies for the anticipated non-responders. Finally, mid-term effects and incidence of disease recurrences after treatment cessation, or complications derived from the TCZ-associated immunosuppression in patients diagnosed with COVID-19 remain to be determined.

In March 2020, the Spanish Agency of Medicines and Medical Devices granted an emergency-use authorization for using TCZ in the setting of COVID-19, and our center developed specific guidelines for treating patients requiring hospital admission. The institutional guidelines included the use of TCZ at an early stage of disease, according to predefined criteria, evaluating clinical, biological and virological outcomes. In this article we describe the short and mid-term effects of early therapy with TCZ and identify predictors of a higher probability of response.

## Results

During the study period, 193 adult patients were admitted with COVID-19. Of them, 89 received TCZ, and 64 met the inclusion criteria and were selected for the analysis; 55 of them had SARS-CoV-2 infection confirmed by RT-PCR. Overall cohort mortality (95% CI) was 5.6 (3–10)%; 4.5 (1.5–11.7)% in patients receiving TCZ, and no deaths occurred in patients meeting the inclusion criteria. Median (Q1–Q3) SOFA score was 1.5 (1–2), and median (Q1–Q3) number of days from illness onset to hospital admission was 10 (7–14). Baseline characteristics of included patients are shown in Table 1. Median (Q1–Q3) age was 62 (56–74) years, and 47 (73%) were male. The majority (61%) of patients had coexisting comorbid diseases, mainly hypertension (34%), cardiovascular disease (14%), diabetes (14%) and chronic obstructive pulmonary disease (COPD) (5%), with median (Q1–Q3) Charlson comorbidity index score of 2 (1–4). Antimicrobial and/or immunomodulatory therapy administered during hospital stay is detailed in Table 1.

**Table 1:** Demographic, clinical and laboratory findings on admission.

Variables^a^	All		Death, ICU admission or SOFA ≥ 3				OR (CI 95%)	*P* value^b^
			Yes		No			
Patients, no	64		15		49		–	–
Male, no. (%)	47	(73)	13	(86)	2	(13)	2.86 (0.57–14.31)	0.317
Age, years	62	(56–74)	77	(68–85)	60	(53–69)	1.10 (1.04–1.16)	0.002
**Coexisting conditions**								
Charlson-CI	2	(1–4)	3	(1–3)	1	(1–2)	1.51 (1.13–2.01)	0.005
Any^c^, no. (%)	39	(61)	11	(73)	28	(57)	2.06 (0.57–7.39)	0.266
Hypertension, no. (%)	22	(34)	7	(47)	15	(31)	1.98 (0.61–6.47)	0.256
Diabetes, no. (%)	9	(14)	5	(33)	4	(8)	5.62 (1.27–24.76)	0.022
Cardiovascular disease^d^, no. (%)	9	(14)	3	(20)	6	(12)	1.79 (0.38–6.33)	0.454
COPD, no. (%)	3	(5)	2	(13)	1	(2)	0.13 (0.01–1.61)	0.072
Days from illness onset	10	(7–14)	9	(4–15)	11	(8–14)	0.94 (0.84–1.06)	0.337
**Vital signs**								
Body temperature, °C	36.5	(36.1–36.9)	36.9	(36.4–37.3)	36.4	(36.1–36.7)	4.64 (1.14–15.23)	0.011
Systolic blood pressure, mmHg	120	(113–134)	135	(123–148)	120	(112–129)	1.06 (1.02–1.11)	0.003
Diastolic blood pressure, mmHg	74	(70–81)	80	(67–87)	74	(70–80)	1.02 (0.97–1.07)	0.419
Mean blood pressure^f^	92	(86–99)	99	(90–104)	91	(83–96)	1.08 (1.01–1.15)	0.032
Supplemental oxygen, no (%)	54	(84)	14	(93)	40	(82)	3.15 (0.36–27.14)	0.296
SpO_2_, percentage	96	(95–97)	95	(94–96)	96	(95–97)	0.64 (0.42–0.98)	0.040
SpO_2_/FIO_2_	346	(337–365)	342	(339–360)	346	(335–365)	0.99 (0.98–1.01)	0.281
SOFA score	1.5	(1–2)	2	(2–2)	1	(1–2)	16.2 (1.99–131.74)	0.009
Bilateral lung infiltrates, no (%)	47	(73)	11	(73)	36	(73)	0.76 (0.19–2.92)	0.695
**Laboratory findings**								
Lymphocyte count, × 10^3^/μL	1.06	(0.82–1.57)	0.99	(0.71–1.29)	1.12	(0.83–1.61)	1.26 (0.84–1.87)	0.250
Neutrophil-to-lymphocyte ratio	2.5	(1.8–5.3)	8.1	(3.7–11.2)	2.4	(1.4–3.3)	1.21 (1.05–1.40)	0.009
C-reactive protein, mg/L	50	(18–91)	85	(52–148)	45	(18–76)	1.01 (0.99–1.01)	0.126
IL-6, pg/mL	85	(39–181)	108	(45–241)	84	(23–159)	1.01 (0.99–1.01)	0.507
d-dimer, μg/mL	0.83	(0.46–1.73)	1.36	(0.7–2.13)	0.61	(0.46–1.19)	0.98 (0.77–1.23)	0.875
Lactate dehydrogenase, U/L	243	(193–297)	280	(232–329)	240	(188–278)	1.01 (1.01–1.01)	0.041
Ferritin, ng/mL	439	(307–634)	448	(323–596)	438	(301–648)	0.99 (0.99–1.01)	0.674
High HS-cardiac troponin I, no. (%)^g^	10	(18)	4	(31)	6	(14)	2.81 (0.65–12.10)	0.164
NT-proBNP, pg/mL	82	(36–188)	85	(10–303)	80	(41–171)	1.00 (0.99–1.01)	0.777
**Concomitant antimicrobial/immunomodulatory drugs, no. (%)**								
HCQ-based combinations	64	(100)	15	(100)	49	(100)	–	–
Azithromycin	60	(94)	14	(93)	46	(94)	1.09 (0.10–11.3)	0.938
Lopinavir/ritonavir	63	(98)	14	(93)	49	(100)	10.2 (0.39–256.1)	0.091
Remdesivir	0	–	–		–		–	–
Interferon-β-1b	11	(17)	4	(27)	7	(14)	0.16 (0.03–0.82)	0.019
Methylprednisolone^e^	11	(17)	2	(13)	9	(18)	1.46 (0.28–7.65)	0.653
**Follow-up**								
SpO_2_/FIO_2_ (48 h)	346	(323–359)	312	(273–354)	350	(339–358)	0.98 (0.97–0.99)	0.032
C-reactive protein (48 h), mg/L	22	(8–44)	46	(32–81)	16	(7–37)	1.02 (1.01–1.03)	0.011
Supplemental oxygen (48 h), no (%)	50	(78)	14	(93)	36	(74)	5.05 (0.60–42.3)	0.135
Supplemental oxygen (7 days), no (%)	43	(67)	14	(93)	29	(59)	9.65 (1.17–79.4)	0.035
Radiological progression	13	(20)	3	(20)	10	(20)	0.97 (0.23–4.19)	0.973
SOFA ≥ 3 during follow-up, no (%)	12		12		–		–	–
ICU admission	3		3		–		–	–
Death	0		–		–		–	–

*ICU* intensive care unit, *Charlson-CI* Charlson Comorbidity Index, *SpO*_*2*_ pulse oximetric saturation, *FIO*_*2*_ fraction of inspired oxygen, *SOFA* Sequential Organ Failure Assessment, *HCQ* hydroxychloroquine, *IL-6* interleukin 6, *NT-proBNP* N-terminal Pro B-type Natriuretic Peptide.

^a^Values expressed as median (interquartile range) unless stated otherwise.

^b^P values are obtained from univariate logistic regression modeling.

^c^Cardiovascular (i.e., hypertension, coronary artery disease, chronic heart failure, cerebrovascular disease and peripheral arterial disease), respiratory (i.e., chronic obstructive pulmonary disease [COPD], asthma), chronic kidney failure, immunosuppression, malignancy, liver cirrhosis, systemic autoimmune disease or diabetes mellitus.

^d^Cardiovascular disease other than hypertension.

^e^Short course methylprednisolone 0.5–1 mg/kg/day divided in 2 intravenous doses for 3 days.

^f^Mean blood pressure was calculated as (2/3·diastolic blood pressure) + (1/3·systolic blood pressure).

^g^Values for HS-cardiac troponin I were available in 57 patients (44 and 13 in favorable and unfavorable groups, respectively). Since they were skewed left, resulting in very few cases with values above upper normal limit and precluding calculation of informative odds ratios and 95% confidence intervals, the variable was categorized with a cut-off value of 0.2 ng/mL.

After therapy with TCZ, 49 (76.6%) patients had a favorable and 15 (23.4%) unfavorable response or adverse outcome, defined as an increase in the SOFA score > 2 measured at 48–72 h or at day 7 (12 patients; 18.8%), ICU admission (3 patients, 4.7%) or death (0 patients). Patients with favorable response to TCZ were younger (60 [53–69] vs 77 [68–85] years, p = 0.002), had a significant lower Charlson comorbidity index (1 [1, 2] vs 3 [1, 3], p = 0.005), lower frequency of diabetes (4 [8%] vs 5 [33%], p = 0.022), lower mean (91 [83–96] vs 99 [90–104] mm Hg, p = 0.032) and systolic (120 [112–129] vs 135 [123–148] mm Hg, p = 0.003) blood pressure, lower axillary temperature, higher pulse oximetry saturation (96 [95–97]% vs 95 [94–96], p = 0.040), lower SOFA score (2 [2–2] vs 1 [1, 2], p = 0.009), lower neutrophil/lymphocyte ratio (NLR) (2.4 [1.4–3.3] vs 8.1 [3.7–11.2], p = 0.009), and lower LDH levels (240 [188–278] vs 280 [232–329] U/L, p = 0.041). Responders to TCZ had received less frequently interferon-β-1b (7 [14%] vs 4 [27%], p = 0.019), with no differences between groups in the frequency of treatment with methylprednisolone. There were no disease recurrences after TCZ interruption, or additional bacterial infections as a complication of therapy during hospitalization or at the 4-week follow-up visit. Among the 55 patients meeting the inclusion criteria with confirmed SARS-CoV-2 infection by RT-PCR, there were 43 (78.18%) responders and 12 (21.82%) non-responders. When both groups were compared, the results were similar to those observed in the 64 patients comprising the entire study sample (see Supplementary Table 1).

Table 2 shows the multivariate logistic regression analysis to identify predictors of response to TCZ. The model showed that male sex (OR 6.70; 95% CI 1.05–42.96), a NLR > 2.55 (OR 4.55; 95% CI 1.03–20), higher SOFA score (OR 6.05; 95% CI 1.27–28.8 per unit increase), higher systolic blood pressure (OR 1.07; 95% CI 1.01–1.14 per mmHg) and higher Charlson comorbidity index (OR 1.35; 95% CI 1.03–1.79 per unit increase), were associated with unfavorable outcome following TCZ administration. In a sensitivity analysis including only the 55 patients with confirmed SARS-CoV-2 infection by RT-PCR, the significant variables in the adjusted multivariate model were a NLR > 2.55 (OR 5.26; 95% CI 1.02–25), higher Charlson comorbidity index (OR 1.56; 95% CI 1.04–2.34) per unit, and higher SOFA score (OR 5.05; 95% CI 1.10–23.24) (Supplementary Table 2).

**Table 2:** Predictors of unfavorable outcome after tocilizumab initiation in multivariate logistic regression analysis.

Baseline variable	OR (95% CI)	P value
Male sex	6.70 (1.05–42.96)	0.045
Charlson-CI, per unit	1.35 (1.03–1.79)	0.032
Systolic blood pressure, per mmHg	1.07 (1.01–1.14)	0.027
SOFA score, per unit	6.05 (1.27–28.8)	0.024
Neutrophil-to-lymphocyte ratio > 2.55	4.55 (1.03–20)	0.045
Platelet-to-d-dimer ratio < 200	3.05 (0.81–11.45)	0.098

Unfavorable response was defined as reaching a SOFA score > 2 during hospital stay, Intensive Care Unit admission or death.

*Charlson-CI* Charlson Comorbidity Index, *SOFA* Sequential Organ Failure Assessment.

**Figure 1:**
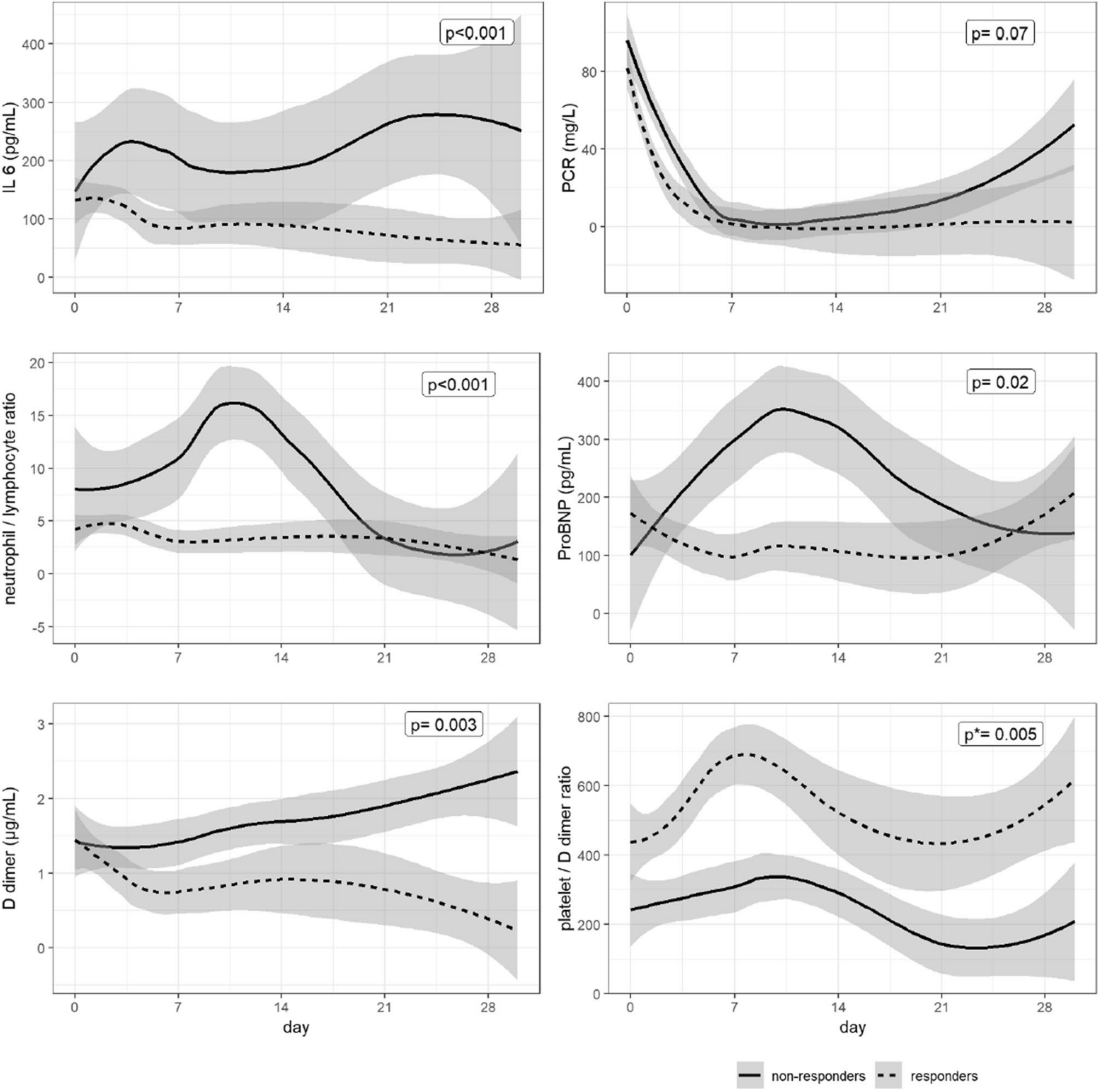
Temporal changes in the levels of several biomarkers in tocilizumab responders and non-responders analyzed through local polynomial regression. P values for the comparison in the temporal trends between responders and non-responders. *P value for the comparison of baseline levels of the biomarker between responders and non-responders. No significant differences observed in the temporal trends between groups. *LOD* limit of detection.

Figure 1 shows the temporal changes of several biomarkers in TCZ responders and non-responders analyzed through local polynomial regression. Non-response to TCZ was associated with an increase in IL-6 not observed in responders (p < 0.001), and the same occurred for the levels of d-dimer (p = 0.003), the NLR (p < 0.001), and the NT-ProBNP (p = 0.02). For the platelet/d-dimer ratio, initial levels were significantly lower in non-responders (p = 0.005), although trends were not significantly different between groups. The CRP levels decreased in both groups, and the decrease tended to be faster in responders to TCZ (p = 0.07).

Table 3 shows ROC analysis to identify plasma biomarkers and clinical variables that, measured at 48 h after TCZ initiation, best discriminated between favorable or unfavorable further clinical outcome. The best predictors among the biomarkers were the CRP, the platelet/d-dimer ratio and the hs-cardiac troponin. At 48 h of TCZ initiation, a CRP value above 25 mg/dL (specificity [SP] 86%; sensitivity [SE] 68%; area under the ROC curve [AUC] [95% CI] 0.78 [0.63–0.92]), a platelet/d-dimer ratio above 343 (SP, 92; SE, 61%; AUC [95% CI] 0.73 [0.59–0.87]) and a hs-cardiac troponin I higher than 0.02 ng/mL (SP, 82%; SE, 58%; AUC [95% CI] 0.74 [0.57–0.91]) significantly predicted an unfavorable subsequent response. A cut-off value > 5 of a composite score including 10 clinical and biological variables (Table 3) showed a specificity of 90% and a sensitivity of 91% to predict adverse outcome, with an AUC (95% CI) of 0.96 (0.91–1.00). A sensitivity analysis including only the 55 patients with microbiologically confirmed infection showed significant AUC [95% CI] for the CRP (0.80 [0.65–0.96]) and the platelet/d-dimer ratio (0.73 [0.59–0.87]), and also for the NLR (0.71 [0.52–0.86]) and LDH (0.73 [0.55–0.90]) (Supplementary Table 3).

**Table 3:** Performance in ROC analysis of serum biomarkers and clinical variables at 48 h of tocilizumab initiation on clinical outcome.

Variable	Absolute variable value at 48 h			
	AUC (95% CI)	Cut-off value	Sp	Sn
C-reactive protein	0.78 (0.63–0.92)	25 mg/L	86	68
IL-6	0.65 (0.42–0.88)	177 pg/mL	63	79
Neutrophil-to-lymphocyte ratio	0.67 (0.48–0.86)	5.3	64	79
Lactate dehydrogenase	0.66 (0.47–0.84)	307 U/L	50	89
HS-cardiac troponin I	0.74 (0.57–0.91)	0.02 ng/mL	82	58
Platelets	0.68 (0.49–0.86)	182 × 10^3^/μL	50	92
Platelet-to-d-dimer ratio	0.73 (0.59–0.87)	343	92	61
SpO_2_	0.81 (0.69–0.93)	96%	93	69
SpO_2_/FIO_2_	0.70 (0.53–0.88)	334	67	79
Systolic blood pressure	0.69 (0.54–0.84)	120 mmHg	88	50
Composite score	0.96 (0.91–1.00)	5.5	90	91
Simplified composite score^a^	0.91 (0.91–0.82)	1.5	83	99

Unfavorable response was defined as reaching a SOFA score > 2 during hospital stay, Intensive Care Unit admission or death.

*IL-6* Interleukin 6, *HS* high-sensitivity, *AUC* area under the ROC curve, *CI* confidence interval, *Sp* specificity (%), *Sn* sensitivity (%).

^a^Composite score using IL-6, platelets, SpO_2_ and SpO_2_/FIO_2_ variables.

**Figure 2:**
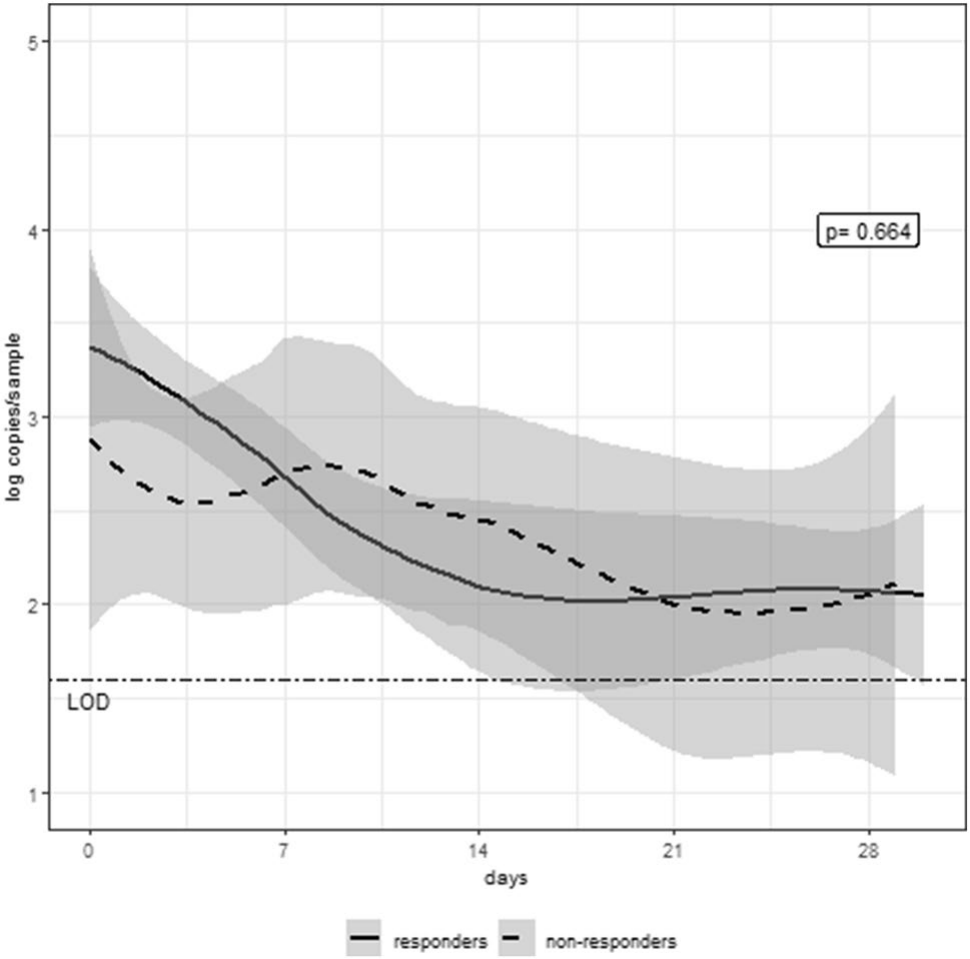
Temporal changes in the levels of SARS-CoV-2 RNA obtained from nasopharyngeal swabs among tocilizumab responders and non-responders analyzed through local polynomial regression. P value for the comparison in the temporal trend between responders and non-responders.

SARS-CoV-2 RNA levels in nasopharyngeal samples at TCZ initiation were not different between responders, who showed a median (Q1–Q3) viral load of 3.89 (2.86–4.59) copies/swab, and non-responders, with 3.48 (2.5–5.249) copies/swab (p = 0.863). Figure 2 shows temporal changes of SARS-CoV-2 RNA obtained from nasopharyngeal swabs among TCZ responders and non-responders analyzed through local polynomial regression. At 1 month after admission, 23/41 (56.10%) of TCZ responders and 3/10 (30%) of non-responders showed undetectable viral load (p = 0.173).

## Discussion

In this cohort of patients admitted with COVID-19 in whom TCZ was used at an earlier stage of disease, there was a low rate of mortality. Previous studies included severely-ill patients, and mortality rates between 10 and 20%^11–13^, with the exception of a study including 20 patients, where no deaths occurred^10^. Our results support that prompt initiation of therapy with TCZ is safe in the short and mid-term, and might be associated with a favorable outcome in patients hospitalized with COVID-19, as reflected by the low overall mortality observed in our entire cohort of patients admitted for COVID-19, which was lower than the 21.8% mortality described in hospitalized patients in Spain (https://www.mscbs.gob.es/en/profesionales/saludPublica/ccayes/alertasActual/nCov-China/situacionActual.htm). We identified early clinical and biological predictors of response to TCZ, which might contribute to selecting alternative strategies for the anticipated non-responders. We found that non-response to TCZ was associated with higher SOFA score and systolic blood pressure on admission, as well as a higher Charlson comorbidity index. Baseline levels and the initial response after TCZ administration of some biomarkers were also useful to predict the clinical outcome; a lower NLR on admission, and lower platelet/d-dimer ratio and hs-cardiac troponin I at 48 h following TCZ initiation were associated with favorable TCZ response. Likewise, the performance of several biomarkers after TCZ initiation revealed to be useful to discriminate among responders and non-responders. Specifically, the levels of IL-6, NLR, d-dimer, NT-ProBNP, and the platelet/d-dimer ratio showed an increase in non-responders that was not observed in responders.

One of the concerns regarding anti-IL-6 therapy is the potential risk of impaired viral infection control. IL-6 regulates the immune response through differentiation of CD4 and CD8 T cells to induce a cytotoxic and cytolytic activity against pathogens^14^. In experimental studies, IL-6 has demonstrated to induce T cell and B cell responses against viruses for viral clearance, the phagocytic activity of macrophages and tissue remodeling^16,17^, and to suppress the replication of viruses like hepatitis B or HIV^14,18^. Therefore, anti-IL-6 therapy could potentially have detrimental consequences if given too early in patients with COVID-19, before the full effect of the antivirals has been established. However, data also exist pointing to a negative influence of IL-6 on viral clearance by inhibition of CD8 T cell response, favoring viral persistence through a synergistic interaction with IL-17^19^, leading to higher viral replication^20^ and higher virulence^21,22^. In our cohort, patients receiving TCZ had a favorable outcome, and viral clearance was not significantly different among those with favorable or unfavorable response, although due to the low number of patients, our study cannot exclude a potential relationship between the worse clinical outcome and a negative effect of TCZ on viral dynamics in the subgroup of non-responders. We neither observed additional bacterial infections as a complication of therapy during hospitalization or at the 30-day follow-up visit.

We analyzed baseline and serial levels of biomarkers of inflammation, coagulation and myocardial function on an every-other-day schedule during hospital stay, and at 1 month after admission. We found that the initial levels of a number of different biomarkers allowed the prediction of subsequent response to TCZ. Lower levels on admission of the NLR were associated with a favorable TCZ response after adjusting for other predicting covariables. This biomarker reflects excessive inflammation and dysregulation of immune cells that play a central role in severity of disease in viral infections^23^, and has been associated with mortality in patients hospitalized with COVID-19^24^. The NLR has shown to be the most important factor for the early-stage prediction of disease progression in patients with COVID-19, and a useful tool for risk stratification^25^. Our findings support a similar central role of the NLR for the prediction of response to TCZ, and also suggest that early initiation of therapy with TCZ before the catastrophic hyper-inflammatory response has been triggered might improve the prognosis of patients with COVID-19.

In addition to baseline levels, we found that the initial response of some biomarkers after TCZ administration also predicted disease outcome. The levels of CRP at 48 h after TCZ infusion with a cut-off value of 25 mg/dL showed a high specificity and moderate-to-high sensitivity to predict favorable subsequent response. The same was observed with a cut-off value of 343 for the platelets/d-dimer ratio and of 0.02 ng/mL for the hs-cardiac troponin I, although for the latter value the sensitivity was fair. The association of biomarkers of coagulation and myocardial function with outcome reflects the multifactorial pathogenesis of COVID-19, and its potential reversibility with anti-IL-6 therapy before the overt disease is manifested. Conversely, further immune-based or antiviral therapy should be considered in addition to TCZ when the initial levels of the biomarkers predict poor response, in order to optimize treatment results.

The performance of several biomarkers after drug administration was another factor contributing to characterize the responders to TCZ. The levels of the inflammation markers IL-6 and the NLR showed an increasing trend during the following days after TCZ initiation in non-responders, and peaked several days afterwards; by contrast, they remained relatively stable in patients with favorable outcome. As a result, in some patients TCZ would not be capable of inhibiting the cytokine storm, and this would be a predictor of poor outcome. IL-6 showed a second peak during the 4^th^ week after TCZ, and the same was seen for CRP, and again only in non-responders, probably reflecting more than one flare of disease in this subset of patients. In addition to inflammation, a biomarker of cardiac disease such as the proBNP also showed a raising trend in non-responders, suggesting that myocardial dysfunction may contribute as well to the lung complications and adverse outcome. The same occurred with D-dimer, an independent predictor of hospital death in patients with COVID-19^26^, that reflects a pro-coagulant state and increased thrombotic risk associated with severe disease in COVID-19^27^, and which only showed a decreasing trend in patients responding to TCZ. The role of IL-6 in venous thromboembolism remains to be well defined. While IL-6 has been associated with the resolution of thrombi through macrophage recruitment and the induction of proteolytic enzymes^28^, other studies have demonstrated an association between the development of deep venous thrombosis and the inflammatory injury of vascular endothelial cells caused by the imbalance of cytokine expression and, particularly, IL-6 was significantly upregulated in clinical and experimental studies of deep venous thrombosis, and blocking IL-6 in mice with neutralizing antibodies inhibited the formation and development of thrombosis^29^.

The Charlson comorbidity index was a predictor of response to TCZ. Our patients had a high frequency of hypertension, followed by cardiovascular diseases, diabetes and COPD, as described in other series^30^. The four comorbidities are included in the Charlson index, and have been associated with higher disease severity in patients with COVID-19^31^. Although the implicated pathogenic mechanisms have not been characterized, in patients with diabetes or underlying cardiovascular disease, the infection could induce additional damage due to direct or indirect effects related to inflammation, hypercoagulable state and hypoxia^32^, which may aggravate the acute respiratory syndrome. The inverse association observed between the Charlson score and the response to TCZ supports the recent results of a nationwide cohort of hospitalized patients with COVID-19, where a greater number of comorbidities was associated with poorer outcome^33^.

Blood pressure showed to be an independent predictor of response to TCZ, and lower levels on admission were associated with a better response to TCZ. SARS-CoV-2 binds to the angiotensin converting enzyme-2 (ACE-2) receptor, and the expression of ACE-2 is increased in patients treated with ACE inhibitors and angiotensin II type-I receptor blockers^34,35^. This may facilitate infection with SARS-CoV-2, and might also lead to higher severity of disease and mortality. Higher systolic blood pressure in our study could be a surrogate marker of hypertension, and might also be associated with raised filling pressures and contribute to pulmonary edema in patients with ARDS. Interestingly, increased levels of hs-troponin I and NT-proBNP were associated with non-response to TCZ, which might suggest that the cytokine-related stress cardiomyopathy might not be reversed by TCZ if it is not promptly initiated. This impaired myocardial function may be aggravated in patients with higher blood pressures and/or hypertensive heart disease.

Limitations of the study are the absence of a comparable control group, and the possibility of a selection bias in patients receiving TCZ due to the observational nature of the study. The small number of non-responders to TCZ does not allow to exclude a role of viral load as a predictive factor, and other potential contributors like the host immune response were not explored in the study. Strengths are the distinctive characteristics of the population included, and the exhaustive sequential analysis of a high number of biomarkers following TCZ administration which, in addition to clinical factors, allowed the characterization of the patients with a higher probability of response.

In conclusion, early therapy with TCZ is safe and it is associated with low mortality. Our results suggest that a prompt use of TCZ before the overwhelming secretion of cytokines has occurred might lead to better patient outcomes, and support confirmatory randomized clinical trials to assess the role of immune modulator therapies at an early stage of COVID-19.

## Methods

From all hospital admissions due to COVID-19 at Hospital Universitario of Elche, Spain, between March 10th and April 17th, 2020, consecutive patients treated with TCZ with an initial SOFA score < 3 were included in the analysis. Patients with confirmed or probable COVID-19 according to European Centre for Disease Prevention and Control criteria (https://www.ecdc.europa.eu/en/covid-19/surveillance/case-definition) were included. Microbiological confirmation was performed through real-time polymerase chain reaction (RT-PCR) from a nasopharyngeal smear sample or a bronchial aspirate.

Patients were managed according to a pre-defined protocol approved by Hospital General Universitario de Elche, consisting in the collection of epidemiological, demographic, and clinical data, as well as serial nasopharyngeal samples for SARS-CoV-2, chest X-ray, and routine blood tests including levels of interleukin 6 (IL-6), ferritin, lactate dehydrogenase (LDH), magnesium, procalcitonin, C-reactive protein (CRP), N-terminal pro b-type natriuretic peptide (NT-proBNP), highly sensitive (hs)-troponin I, d-dimer, and coagulation tests. Comorbidities and the Charlson comorbidity index were collected on admission. Blood samples for lab tests, electrocardiogram (EKG), chest X-ray and nasopharyngeal samples for SARS-CoV-2 were obtained at different time points during hospital stay. In addition to SARS-CoV-2, PCR analysis for influenza A and B viruses and for respiratory syncytial virus (RSV) were also performed from nasopharyngeal samples upon admission, as well as pneumococcal and *Legionella* antigens from a urine sample, and serological test for HIV, HCV and HBV, and the Quantiferon test.

As stated before, therapy for COVID-19 was given following institutional guidelines of Hospital General Universitario de Elche. Patients received antimicrobial and/or immunomodulatory therapy containing lopinavir/ritonavir, hydroxychloroquine, azithromycin or interferon-β-1b ± methylprednisolone. According to guidelines, tocilizumab (TCZ) was added to initial therapy on admission if any of the following criteria were met: a CURB-65 > 2; oxygen saturation < 93%; respiratory frequency > 30 per min; a chest radiograph with bilateral multilobar infiltrates; one of the following biological markers: d-dimer ≥ 0.7 µg/L, IL-6 ≥ 40 pg/mL, lymphocytes < 800 × 10^9^/L, ferritin ≥ 700 µg/L, fibrinogen > 700 mg/dL or PCR > 25 mg/L. The dose of TCZ was 600 mg iv if the weight was ≥ 75 kg or 400 mg if the weight was < 75 kg. The patients were reevaluated on the following 24 h. Response to therapy was defined as resolution of fever, improvement in tachypnea, improvement in oxygen saturation by at least 5%, decrease in CRP of at least 25%, or no radiological progression 24 h after TCZ administration. Non-response was defined by the absence of any of 24 h response criteria, or an increase in the SOFA score > 2 measured at 48–72 h or at day 7 after TCZ, ICU admission or death. If no clinical response was achieved at 24 h after TCZ administration, a second dose of TCZ (400 mg) or intravenous corticosteroids were administered. Contraindications to the use of TCZ drug were a neutrophil count < 500 × 10^9^/L, platelets ≤ 50,000 × 10^9^/L, AST/ALT > 5 × upper normal limit and documented sepsis by other pathogens. The protocol was approved by the Ethical Committee of the Hospital General Universitario de Elche (Spain) as part of the COVID-19@Spain study. Informed consent was obtained from all subjects.

For RNA extraction and RT-PCR analysis for SARS-CoV-2, nasal and oropharyngeal flock swabs were placed together into 3 mL transport medium (VICUM, Deltalab, Rubí, Spain). Viral RNA was extracted from 350 µL of the medium using the RNeasy Mini Kit (QIAGEN, Hilden, Germany) according to the manufacturer's instructions and eluted in a final 50 µL nucleic acid elution sample. Eight µL RNA was used for detection of SARS-CoV-2 by RT-PCR, with a commercially available kit (ALLPLEX 2019-nCoV Assay, Seegene, Seoul, Korea) which targeted the E, RdRP and N genes. Viral load measurements of nasal/throat samples (log10 copies/sample) were performed with a standard curve of ten-fold serial dilutions from an in vitro RNA transcript (MACROGEN, Seoul, Korea). Assay procedure was carried out in accordance with the manufacturer’s protocol, in a CFX96 real-time thermocycler (BIO-RAD, California, USA). The success of RNA extraction and PCR were assessed by the internal control included in the kit and negative and positive controls were used in each assay.

### Statistical analyses

Continuous variables are expressed as median ± 25th and 75th percentiles (Q1, Q3), and categorical variables as percentages. Wilcoxon or Student’s t-test were used to compare continuous variables, and the chi-square or Fisher’s exact test for categorical variables comparison. Normality of data samples was assessed using Shapiro–Wilk test. Odds ratios (OR) were estimated through generalized linear models with binary response and logit link function. To represent the temporal changes of the biomarkers levels, local polynomial regression models were employed using weighted least squares to estimate the performance of each biomarker according to the day since TZC was initiated. Differences in temporal trends between biomarkers were analyzed through linear mixed models in which an interaction term of day since TCZ initiation and response was included. Confidence intervals for the area under the receiver operating characteristic curve (AUC of the ROC curve) were calculated through stratified bootstrap replicates with 2000 attempts. The cut-off values with the best sensitivity and specificity were selected. Statistical analysis was performed using R-project version 3.6.2 (2019-12-12) and the libraries ggplot2 for the figures^36^.

## Supplementary information

Supplementary Tables.

## Data Availability

The datasets generated and/or analyzed during the current study are available from the corresponding author on reasonable request.
