# Supplementary material for: Preemptive interleukin-6 blockade in patients with COVID-19
Source: Sci Rep. 2020 Oct 8;10:16826. doi: 10.1038/s41598-020-74001-3 (PMC7545205; doi:10.1038/s41598-020-74001-3)
Supplement: Supplementary file 1 — Supplementary Tables. [file 41598_2020_74001_MOESM1_ESM.docx]

**Supplemental Table 1. Demographic, clinical and laboratory findings on admission in patients with confirmed infection by RT-PCR**

|  |  | **All** | |  | **Death, ICU admission or SOFA≥3** | | | | | | | |
| --- | --- | --- | --- | --- | --- | --- | --- | --- | --- | --- | --- | --- |
| **Variables^&^** |  |  | |  | **Yes** | |  | **No** | |  | **OR (CI 95%)** | ***P* value^§^** |
| **Patients**, no. |  | **55** |  |  | 12 |  |  | 43 |  |  | - | - |
| **Male**, no. (%) |  | **40** | **(73)** |  | 10 | (83) |  | 30 | (70) |  | 2.17 (0.42-11.30) | 0.359 |
| **Age**, years |  | **62** | **(56-74)** |  | 76 | (68-85) |  | 60 | (53-69) |  | 1.10 (1.03-1.17) | 0.004 |
| **Coexisting conditions** |  |  |  |  |  |  |  |  |  |  |  |  |
| Charlson-CI |  | **2** | **(1-4)** |  | 5 | (2-5.5) |  | 2 | (0.5-3) |  | 1.67 (1.16-2.41) | 0.006 |
| Any^#^, no. (%) |  | **33** | **(60)** |  | 25 | (58) |  | 8 | (67) |  | 1.44 (0.37-5.24) | 0.595 |
| Hypertension, no. (%) |  | **18** | **(33)** |  | 5 | (41) |  | 13 | (30) |  | 1.98 (0.61-6.47) | 0.256 |
| Diabetes, no. (%) |  | **8** | **(15)** |  | 4 | (33) |  | 4 | (9) |  | 4.86 (1.01-26.70) | 0.049 |
| Cardiovascular disease*, no. (%) |  | **9** | **(16)** |  | 3 | (25) |  | 6 | (14) |  | 2.06 (0.43-9.84) | 0.367 |
| COPD, no. (%) |  | **3** | **(6)** |  | 2 | (17) |  | 1 | (2) |  | 7.53 (0.55-252) | 0.117 |
| **Days from illness onset** |  | **11** | **(8-14)** |  | 9 | (5-14) |  | 11 | (9-14) |  | 0.95 (0.83-1.08) | 0.401 |
| **Vital signs** |  |  |  |  |  |  |  |  |  |  |  |  |
| Body temperature, ºC |  | **36.5** | **(36.1-36.9)** |  | 37.0 | (36.5-37.3) |  | 36.4 | (36.1-36.8) |  | 3.80 (1.07-13.44) | 0.039 |
| Systolic blood pressure, mmHg |  | **120** | **(115-135)** |  | 135 | (125-148) |  | 120 | (114-131) |  | 1.06 (1.01-1.11) | 0.012 |
| Diastolic blood pressure, mmHg |  | **75** | **(70-81)** |  | 81 | (68-92) |  | 75 | (70-81) |  | 1.03 (0.98-1.09) | 0.266 |
| Mean blood pressure^£^ |  | **93** | **(88-99)** |  | 101 | (88-104) |  | 93 | (88-97) |  | 1.08 (0.99-1.16) | 0.077 |
| Supplemental oxygen, no (%) |  | **45** | **(82)** |  | 11 | (92) |  | 34 | (79) |  | 2.91 (0.33-25.62) | 0.340 |
| SpO_2_, percentage |  | **97** | **(95-97)** |  | 95 | (94-96) |  | 97 | (95-96) |  | 0.61 (0.39-0.95) | 0.028 |
| SpO_2_/FIO_2_ |  | **346** | **(337-365)** |  | 343 | (333-344) |  | 346 | (338-367) |  | 0.99 (0.98-1.01) | 0.162 |
| SOFA score |  | **1** | **(1-2)** |  | 2 | (2-2) |  | 1 | (1-2) |  | 17.60 (2.10-147.8) | 0.008 |
| **Bilateral lung infiltrates**, no (%) |  | **42** | **(76)** |  | 10 | (83) |  | 32 | (74) |  | 1.71 (0.32-9.09) | 0.524 |
| **Laboratory findings** |  |  |  |  |  |  |  |  |  |  |  |  |
| Lymphocyte count, x10^3^/𝜇L |  | **1.04** | **(0.82-1.53)** |  | 0.86 | (0.69-1.15) |  | 1.12 | (0.84-1.65) |  | 0.67 (0.22-2.06) | 0.485 |
| Neutrophil-to-lymphocyte ratio |  | **2.51** | **(1.61-7.04)** |  | 8.25 | (4.40-11.62) |  | 2.39 | (139-3.19) |  | 1.27 (1.07-1.49) | 0.006 |
| C-reactive protein, mg/L |  | **46** | **(18-89)** |  | 105 | (56-155) |  | 30 | (17-73) |  | 1.01 (0.99-1.02) | 0.075 |
| IL-6, pg/mL |  | **71** | **(26-159)** |  | 69 | (43-202) |  | 71 | (19-155) |  | 1.01 (0.99-1.01) | 0.630 |
| D-dimer, 𝜇g/mL |  | **0.61** | **(0.45-1.52)** |  | 1.37 | (0.78-2.14) |  | 0.59 | (0.45-1.14) |  | 0.99 (0.78-1.24) | 0.896 |
| Lactate dehydrogenase, U/L |  | **238** | **(197-321)** |  | 306 | (233-414) |  | 234 | (185-284) |  | 1.01 (1.01-1.02) | 0.012 |
| Ferritin, ng/mL |  | **448** | **(321-634)** |  | 523 | (328-615) |  | 438 | (316-638) |  | 1.00 (0.99-1.01) | 0.981 |
| HS-cardiac troponin I, ng/mL^¥^ |  | **7** | **(14)** |  | 3 | (25) |  | 4 | (11) |  | 3.18 (0.59-17.16) | 0.177 |
| NT-proBNP, pg/mL |  | **79** | **(36-169)** |  | 35 | (7-231) |  | 80 | (42-169) |  | 1.00 (0.99-1.01) | 0.862 |
| **Concomitant antimicrobial / immunomodulatory drugs**, no. (%) |  |  |  |  |  |  |  |  |  |  |  |  |
| HCQ-based combinations |  | **55** | **(100)** |  | 12 | (100) |  | 43 | (100) |  | - | - |
| Azithromycin |  | **53** | **(96)** |  | 12 | (100) |  | 41 | (95) |  | 1.56 (0.06-33.48) | 0.450 |
| Lopinavir/ritonavir |  | **54** | **(98)** |  | 11 | (92) |  | 43 | (100) |  | 11.34 (0.43-297.36) | 0.058 |
| Remdesivir |  | **0** |  |  | - |  |  | - |  |  | - | - |
| Interferon-β-1b |  | **10** | **(18)** | 3 | (25) |  |  | 7 | (16) |  | 1.71 (0.37-7.97) | 0.490 |
| Methylprednisolone^&^ |  | **12** | **(22)** | 5 | (42) |  |  | 7 | (16) |  | 0.27 (0.07-1.10) | 0.062 |
| **Follow-up** |  |  |  |  |  |  |  |  |  |  |  |  |
| SpO_2_/FIO_2_ (48 hours) |  | **97** | **(95-98)** |  | 95 | (94-96) |  | 98 | (96-98) |  | 0.61 (0.44-0.86) | 0.004 |
| C-reactive protein (48 hours), mg/L |  | **19** | **(7-39)** |  | 52 | (29-86) |  | 15 | (7-36) |  | 1.02 (1.01-1.04) | 0.011 |
| Supplemental oxygen (48 hours), no (%) |  | **41** | **(75)** |  | 11 | (92) |  | 30 | (70) |  | 4.77 (0.56-40.84) | 0.154 |
| Supplemental oxygen (7 days), no (%) |  | **36** | **(66)** |  | 11 | (92) |  | 25 | (58) |  | 7.92 (0.94-66.97) | 0.057 |
| Radiological progression |  | **10** | **(18)** |  | 1 | (8) |  | 9 | (21) |  | (0.34 0.04-3.02) | 0.335 |
| SOFA ≥3 during follow-up, no (%) |  | **12** |  |  | 12 |  |  | - |  |  | - | - |
| ICU admission |  | **3** |  |  | 3 |  |  | - |  |  | - | - |
| Death |  | **0** |  |  | - |  |  | - |  |  | - | - |

^&^Values expressed as median (interquartile range) unless stated otherwise. **^§^**P values are obtained from univariate logistic regression modeling. ^#^Cardiovascular (i.e., hypertension, coronary artery disease, chronic heart failure, cerebrovascular disease and peripheral arterial disease), respiratory (i.e., chronic obstructive pulmonary disease [COPD], asthma), chronic kidney failure, immunosuppression, malignancy, liver cirrhosis, systemic autoimmune disease or diabetes mellitus. *Cardiovascular disease other than hypertension. ^&^Short-course methylprednisolone 0.5-1 mg/kg/day divided in 2 intravenous doses for 3 days. ^£^Mean arterial blood pressure was calculated as (2/3·diastolic blood pressure)+ (1/3·systolic blood pressure). ^¥^ Values for HS-cardiac troponin I were available in 57 patients (44 and 13 in favorable and unfavorable groups, respectively). Since they were skewed left, resulting in very few cases with values above upper normal limit and precluding calculation of informative odds ratios and 95% confidence intervals, the variable was categorized with a cut-off value of 0.2 ng/mL. ICU, intensive care unit; Charlson-CI, Charlson Comorbidity Index; SpO_2_, pulse oximetric saturation; FIO_2_, fraction of inspired oxygen; SOFA, Sequential Organ Failure Assessment; HCQ, hydroxychloroquine; IL-6, interleukin 6; NT-proBNP, N-terminal Pro B-type Natriuretic Peptide; RT-PCR, real-time polymerase chain reaction.

**Supplemental Table 2. Predictors of unfavorable outcome after tocilizumab initiation in multivariate logistic regression analysis in patients with confirmed infection by RT-PCR**

| **Baseline variable** |  | **OR (95% CI)** | **P value** |
| --- | --- | --- | --- |
| **Male sex** |  | 3.62 (0.51-25.76) | 0.199 |
| **Charlson-CI**, per unit |  | 1.56 (1.04-2.34) | 0.034 |
| **Systolic blood pressure**, per mmHg |  | 1.05 (0.99-1.12) | 0.094 |
| **SOFA score**, per unit |  | 5.05 (1.10-23.24) | 0.038 |
| **Higher neutrophil-to-lymphocyte ratio>2.55** |  | 5.26 (1.02-25) | 0.039 |
| **Lower Platelet-to-D-dimer ratio^&^** |  | 2.51 (0.64-9.89) | 0.188 |

Unfavorable response was defined as reaching a SOFA score >2 during hospital stay, Intensive Care Unit admission or death. Charlson-CI, Charlson Comorbidity Index; SOFA, Sequential Organ Failure Assessment; RT-PCR, real-time polymerase chain reaction

**Supplemental Table 3. Performance in ROC analysis of serum biomarkers and clinical variables at 48 hours of tocilizumab initiation on clinical outcome in patients with confirmed infection by RT-PCR.**

|  |  | **Absolute variable value at 48 hours** | | | |
| --- | --- | --- | --- | --- | --- |
| **Variable** |  | **AUC (95% CI)** | **Cut-off value** | **Sp** | **Sn** |
| C-reactive protein |  | 0.80 (0.65-0.96) | 26 mg/L | 73 | 83 |
| IL-6 |  | 0.63 (0.37-0.89) | 175 pg/mL | 79 | 67 |
| Neutrophil-to-lymphocyte ratio |  | 0.71 (0.52-0.86) | 3.9 | 73 | 75 |
| Lactate dehydrogenase |  | 0.73 (0.55-0.90) | 307 U/L | 87 | 58 |
| HS-cardiac troponin I |  | 0.69 (0.49-0.89) | 0.02 ng/mL | 53 | 78 |
| Platelets |  | 0.67 (0.46-0.87) | 182 x10^3^/𝜇L | 90 | 50 |
| Platelet-to-D-dimer ratio |  | 0.77 (0.63-0.91) | 343 | 68 | 92 |
| SpO_2_ |  | 0.83 (0.71-0.96) | 96.5 % | 74 | 92 |
| SpO_2_/FIO_2_ |  | 0.79 (0.61-0.96) | 334 | 81 | 83 |
| Systolic blood pressure |  | 0.71 (0.55-0.86) | 120 mmHg | 48 | 92 |
| **Composite score** |  | 0.96 (0.91-1.00) | 3.5 | 82 | 100 |
| **Simplified Composite score*** |  | 0.93 (0.86-1.00) | 1.5 | 85 | 100 |

Unfavorable response was defined as reaching a SOFA score >2 during hospital stay, Intensive Care Unit admission or death.IL-6, Interleukin 6; HS, High-sensitivity; AUC, Area under the ROC curve; CI, Confidence interval; Sp, Specificity (%); Sn, Sensitivity (%). * Composite score using IL-6, platelets, SpO2 and SpO2/FIO2 variables
